# Dielectric tetrahedrons as terahertz resonators switched from perfect absorber to reflector

**DOI:** 10.1038/s41598-020-74252-0

**Published:** 2020-10-13

**Authors:** Haosheng Chen, Chenchen Zhou, Yongjian Li, Shuaishuai Liang, Jiang Li

**Affiliations:** 1 State Key Laboratory of Tribology, Tsinghua University, Beijing, 100084 China; 2 School of Mechanical Engineering, University of Science and Technology Beijing, Beijing, 100083 China

**Keywords:** Metamaterials, Materials for optics

## Abstract

Tetrahedrons are basic building blocks in natural and artificial materials, while the terahertz response of micro tetrahedrons has been little explored. Here we fabricate subwavelength ceramic tetrahedrons for use in the terahertz frequency range, and find that the three-dimensional geometry significantly affects their terahertz properties. The transmission spectra are independent of the orientation of the tetrahedrons, while the first magnetic resonance disappears in the reflection spectra when an upright tetrahedron is flipped upside down on the metallic substrate, which changes it from a perfect absorber to a perfect reflector. This is attributed to the destructive interference between two magnetic dipoles induced respectively by the incident and the reflected wave. The study brings new insights in the materials design with 3D building blocks to realize more interesting and exotic terahertz properties.

## Introduction

Metamaterials with subwavelength building blocks have exotic electromagnetic (EM) properties^1–3^, such as negative magnetic permeability, negative electric permittivity and negative refraction index, which have been demonstrated in various applications, e.g. cloaking^4–6^, magnetic mirrors^7^, perfect absorbers^8,9^ and perfect reflectors^10^. Such properties can be engineered by tailoring materials, shapes and sizes of building blocks^11,12^. Both metallic and dielectric building blocks with different shapes^1–3^, such as cubes, disks and planar patterns from lithography, have been studied from microwave to visible wavelengths, while asymmetric three-dimensional (3D) geometries have shown different features in terahertz applications^13,14^. Therefore, the roles of 3D building blocks in the exotic EM properties in terahertz band (wavelength $30\,{\upmu }\hbox {m}{-}3\hbox { mm}$)^15^ raise wide interests accordingly^1,16^.

Tetrahedrons have the simplest 3D geometry^17^. They are not only basic components of natural materials, but also building blocks to form complex structures^17,18^. On one hand, a regular tetrahedron is highly symmetric, where all faces are of the same shape and size. Hence, there is an equal chance of landing on any of its four faces as the dice, which provides a promising method other than lithography to make large surface metamaterial^19^ with randomly distributed tetrahedrons. On the other hand, tetrahedrons at the rest position do not have a symmetric plane parallel to the bottom face, so an upside-down flip of the tetrahedrons changes the surface structure on the substrate, which is a feature totally different from spheres, cubes, disks and planar patterns. However, the EM properties of dielectric tetrahedrons in terahertz frequency range have been little explored, because the requirements of high permittivity materials and the consequent difficulties in fabrication of micro-size dielectric tetrahedrons bring great challenges. Although there are a few studies on the synthesize and the assembly of nano metallic triangular pyramids^14^ and the tetrahedral dices in millimeter scale^18^, the fabrication of regular tetrahedrons in the scale of subwavelength of terahertz frequency range has not been realized, so the terahertz properties of tetrahedrons are still unclear.

In this work, we fabricate zirconia ceramic tetrahedrons in micro size, and investigate terahertz responses of the upright tetrahedrons and their upside-down counterparts with both simulations and experiments. A special feature is found in the reflection spectra that a simple upside-down flip switches the tetrahedron from a perfect absorber to a perfect reflector at the first magnetic resonant frequency, which is attributed to the destructive interference effect caused by the orientation change of the asymmetric geometry.

## Results

### Disappearance of the first resonance in reflection spectra of upside-down tetrahedron

**Figure 1:**
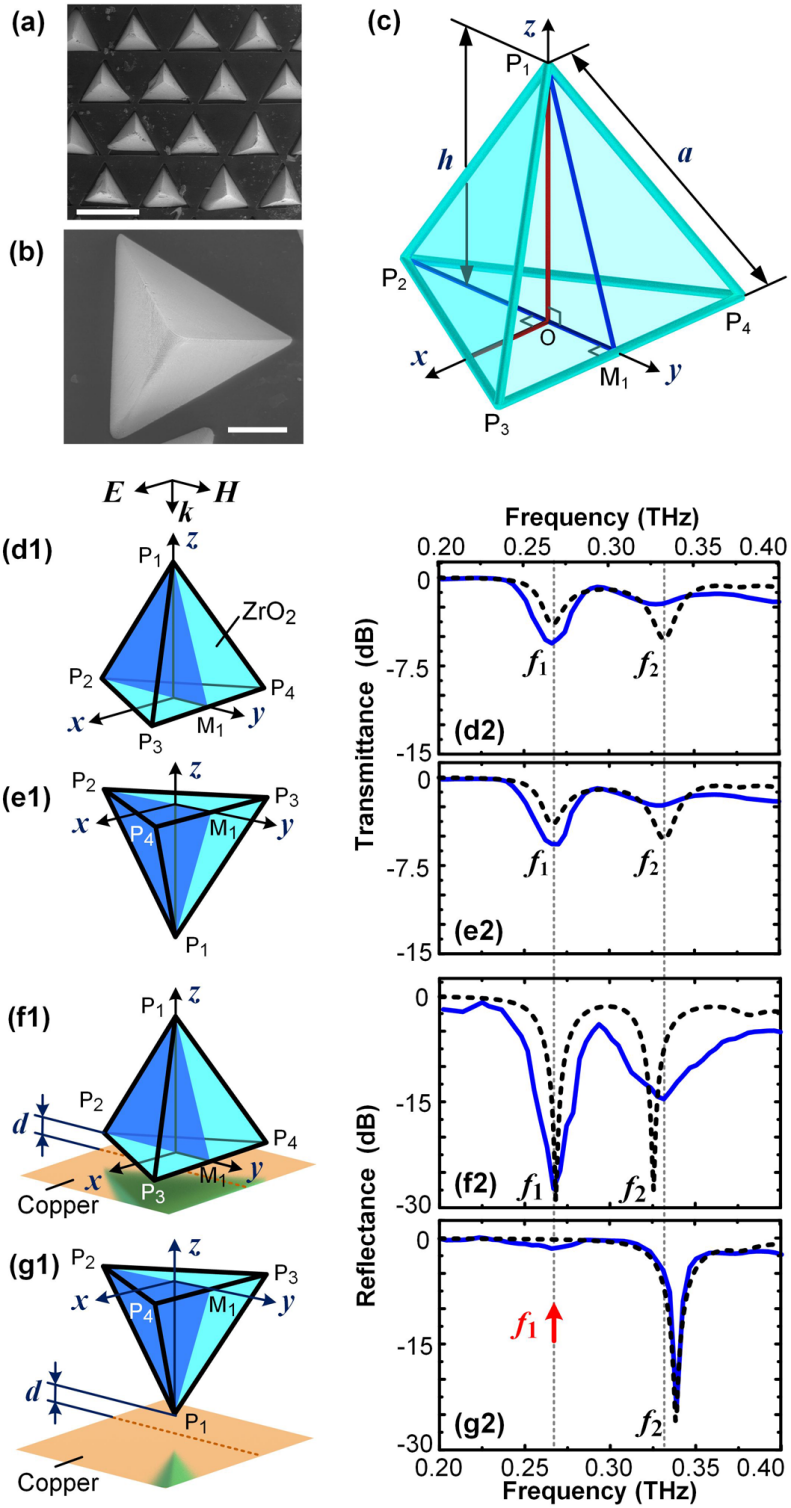
Dielectric tetrahedrons and their responses to the terahertz waves. (**a**) SEM image of zirconia ceramic tetrahedrons in a hexagonal array, where the scale bar is $500\,{\upmu }\hbox {m}$. (**b**) SEM image of an enlarged upright tetrahedron at the rest position, where the scale bar is $100\,{\upmu }\hbox {m}$. (**c**) Schematics of the 3D geometry and the dimensions of a tetrahedron. The six edges are of the same length $a = 355\,{\upmu }\hbox {m}$, while the height, i.e. the length of line segment $\hbox {P}_{1}\hbox {O}$, is $h = 290\,{\upmu }\hbox {m}$. Point $\hbox {M}_{1}$ is the midpoint of the edge $\hbox {P}_{3}\hbox {P}_{4}$, so the cross section triangle ${\Delta }\hbox {P}_{1}\hbox {P}_{2}\hbox {M}_{1}$ is a symmetric plane of the tetrahedron, and it is in the $y{-}z$ plane. (**d1**) Schematics of an upright tetrahedrons in the transmission measurement, illustrated with isometric projection. (**d2**) The simulation and experimental results of the transmission spectra of (**d1**). Black dashed line represents the simulation result, while the blue continuous line represents the experimental result. (**e1**,**e2**) Schematics of an upside-down tetrahedron in the transmission measurement and the corresponding transmission spectra. (**f1**,**f2**) Schematics of an upright tetrahedron over a copper substrate in the reflection measurement and the corresponding reflection spectra. The visible reflection of the tetrahedron on the copper surface indicates that there is a separation *d* between the bottom of the tetrahedron and the copper surface. (**g1**,**g2**) Schematics of an upside-down tetrahedron over a copper substrate in the reflection measurement and the corresponding reflection spectra.

$\hbox {ZrO}_{2}$ ceramic tetrahedrons with a side length $a = 355\,{\upmu }\hbox {m}$ are fabricated as described in detail in Methods. The tetrahedron always rests upright on a substrate with one face at the bottom, as shown in Fig. 1a,b; when it is flipped vertically, an upside-down tetrahedron with its bottom surface on top is obtained. The tetrahedron has four equilateral triangular faces, six straight edges with the same length *a*, and four vertices $\hbox {P}_{1} - \hbox {P}_{4}$, as shown in Fig. 1c. Point $\hbox {M}_{1}$ is the midpoint of the line segment $\hbox {P}_{3}\hbox {P}_{4}$, while point O is the centroid of the bottom triangle ${\Delta }\hbox {P}_{2}\hbox {P}_{3}\hbox {P}_{4}$. Therefore, the line segment $\hbox {OP}_{1}$ is the height of the tetrahedron, which is $h = (2/3)^{1/2}a = 290\,{\upmu }\hbox {m}$. The triangle ${\Delta }\hbox {P}_{2}\hbox {P}_{3}\hbox {P}_{4}$ is in the $x{-}y$ plane, while the triangle ${\Delta }\hbox {P}_{1}\hbox {P}_{2}\hbox {M}_{1}$ is in the $y{-}z$ plane, which is the symmetric plane of the tetrahedron. As $\hbox {ZrO}_{2}$ ceramic has a high permittivity $\varepsilon _{\mathrm{r}} = 32.5$ and a low loss factor of 0.05 in terahertz band^20,21^, it is suitable to be used in terahertz applications. The terahertz responses of four different cases are investigated with experimental measurements and numerical simulations. Two transmission cases of the upright tetrahedrons and the upside-down tetrahedrons are shown in Fig. 1d,e, respectively. Two reflection cases of the upright tetrahedrons and the upside-down tetrahedrons are shown in Fig. 1f,g, respectively, where a metallic copper substrate is placed under the tetrahedrons with the separation $d = 40\,{\upmu }\hbox {m}$. In all four cases, the incident wave (*k*) is normal to the *x*-*y* plane, with the electric field (*E*) polarized along the *x* axis and the magnetic field (*H*) polarized along the *y* axis. When the tetrahedron is flipped upside down, the triangle ${\Delta }\hbox {P}_{1}\hbox {P}_{2}\hbox {M}_{1}$ remains in the $y{-}z$ plane. The details of the experiments and the simulations are described in Methods.

Although the upright tetrahedron and its upside-down counterpart look totally different from the wave propagation direction, as shown in Fig. 1d1,e1, they have identical transmission spectra. The first two resonant frequencies for both cases are found at $f_{1} = 0.27\hbox { THz}$ ($\lambda _{1} = 1.1\hbox { mm}$) and $f_{2} = 0.33\hbox { THz}$ ($\lambda _{2} = 0.9\hbox { mm}$), respectively, as show in Fig. 1d2,e2, so the tetrahedrons are subwavelength particles. For the subwavelength dielectric spheres and cubes, their first resonant frequencies in the transmission spectra are approximately the same as those in their corresponding reflection spectra with a metallic substrate^20,22^, so we expect that the reflection spectra of the upright tetrahedron and its upside-down counterpart, as shown in Fig. 1f1,g1, should have approximately the same resonant frequencies. However, they are significantly different. Although the first two resonant frequencies of the upright tetrahedron are approximately the same as $f_{1}$ and $f_{2}$ in its transmission spectra, as shown in Fig. 1f2, the first resonance of the upside-down tetrahedron at $f_{1}$ disappears, as shown in Fig. 1g2.

The blue continuous lines in the spectra in Fig. 1d2–g2 represent the experimental results, while the black dashed lines represent the simulation results, and the simulation results of the resonant frequencies match well with the experimental results. The unit-cell models for the simulations are established based on the experimental setup, as shown in Supplementary Fig. S1. In the experiments, the upright $\hbox {ZrO}_{2}$ tetrahedrons are arranged in a hexagonal lattice, as shown in Fig. 1a, with a periodicity of $690\,{\upmu }\hbox {m}$ to avoid the interaction between the tetrahedrons, and then embedded in a thin poly-dimethylsiloxane (PDMS) film, which has a negligible permittivity^20^ in terahertz band. The fabrication of the tetrahedron-embedded PDMS film is introduced in Methods. The resultant film has a thickness of $370\,{\upmu }\hbox {m}$, and a simple flip of the film realizes the array of upside-down tetrahedrons directly. Consequently, the film is used as the sample for both transmission experiments of the upright and the upside-down tetrahedrons. In addition, as the PDMS film can be reversibly adhered to a smooth copper surface, it is adhered to a copper surface with different sides for the reflection spectra measurements. Therefore, the same tetrahedron-embedded PDMS film is used in the experiments of all four cases in Fig. 1d–g, which indicates that the difference between the reflection spectra does not result from the difference between the experimental samples.

Compared with the wavelength $\lambda _{1}$ at the first resonant frequency $f_{1}$, the height of the tetrahedrons, $h = 290\,{\upmu }\hbox {m}$, is small enough for the scatter wave to be ignored^20^. In the reflection experiments, as the incident wave is not able to penetrate through the copper layer, the transmission through the copper layer is also ignored^22^. Thus, the incident wave is either reflected or absorbed. Consequently, the reflectance of the upright tetrahedrons on the copper substrate is approximately –30 dB (3 %) at $f_{1}$, which indicates the incident wave is almost entirely absorbed, as shown in Fig. 1f2; while the reflectance of the upside-down tetrahedrons on the copper substrate is approximately 0 dB (100 %), which indicates the incident wave is almost entirely reflected, as shown in Fig. 1g2. The results indicate that the array of the tetrahedrons can be switched between a perfect absorber and a perfect reflector at $f_{1}$ by a simple upside-down flip on the copper layer without changing the thickness of the whole structure. For the terahertz metamaterial composed of planner metallic ring-type resonators^23^, a split-ring resonator can be switched to a closed ring resonator by optical excitation of silicon islands strategically placed in the split gap, which can consequently reconfigure the resonance modes. However, for the metamaterials composed of dielectric particles, the disappearance of the absorption dip at the first resonance due to the upside-down flip of particles has not been reported in previous studies with the typical shapes of spheres^20^, cubes^19,22^, disks^24^ and planar patterns^25^.

### Interference between two magnetic dipoles in the tetrahedron

We then focus on the terahertz response of the tetrahedrons at the first resonant frequency, $f_{1}$, to investigate the disappearance of the first resonance in reflection spectra.

**Figure 2:**
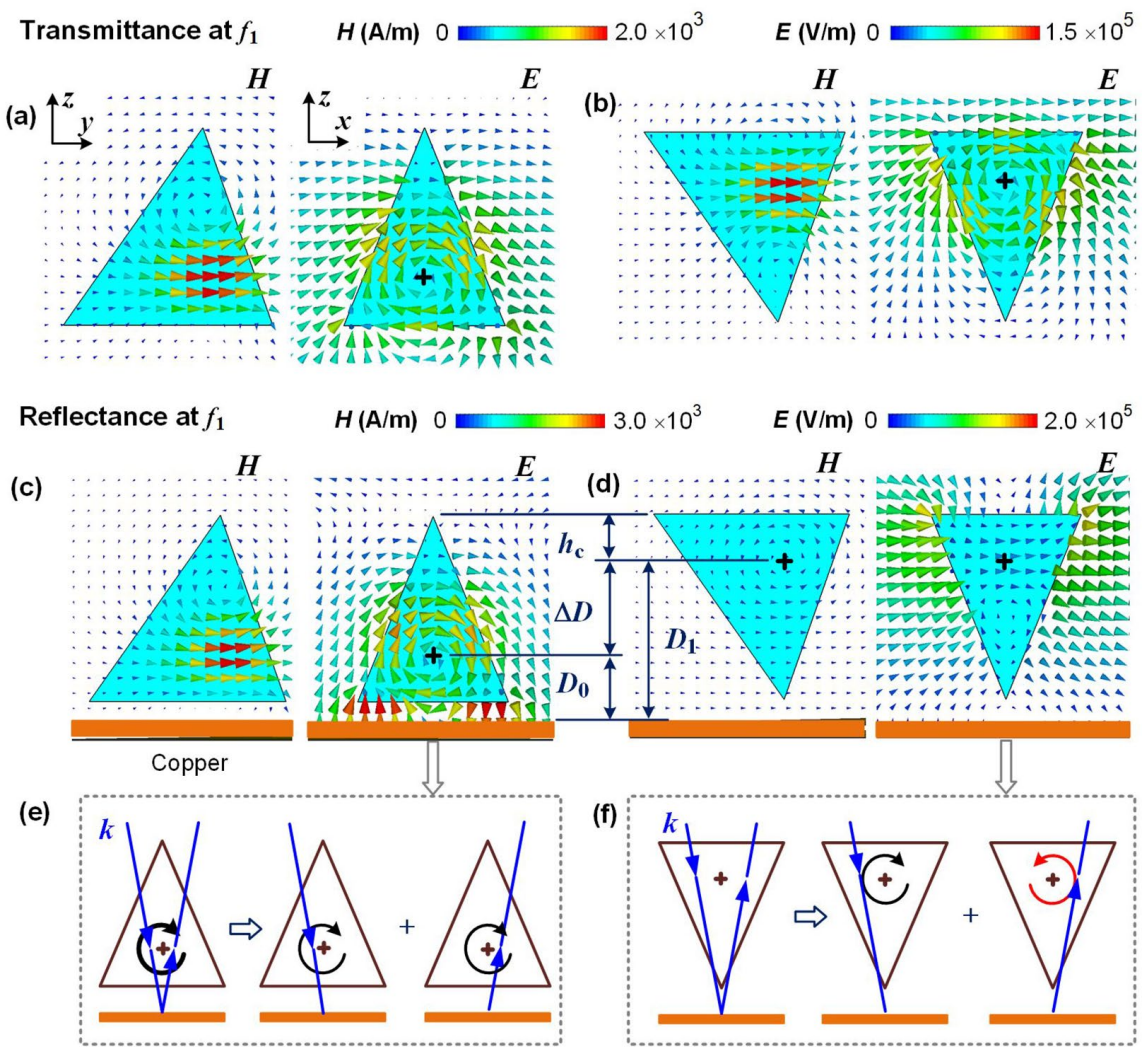
Distribution of the EM fields in the dielectric tetrahedrons at $f_{1} = 0.27\hbox { THz}$. (**a**,**b**) EM fields of the transmission responses in the upright and the upside-down tetrahedrons, respectively. The black cross represents the centroid of the tetrahedron. (**c**,**d**) EM fields of the reflection responses of the upright tetrahedron and the upside-down tetrahedrons on a copper layer, respectively. (**e**,**f**) Schematics of the hypothetic models of the electric fields for the reflection of the upright and the upside-down tetrahedrons on a copper substrate, respectively. The incident wave *k* is approximately normal to the reflective copper layer, and the circular line with an arrow represents the circular displacement current of the magnetic dipole.

According to the Mie resonance theory, the first resonance of subwavelength dielectric spheres or cubes is a magnetic dipole resonance^3^. Here, both the upright and the upside-down tetrahedrons are almost equivalent to a magnetic dipole at the centroid, as shown in the distributions of the magnetic and the electric fields at $f_{1}$ of the transmission spectra in Fig. 2a,b. In both cases, the circular displacement current wraps around the centroid of the tetrahedron in the $x{-}z$ plane, which indicates that a magnetic dipole is induced along the *y* direction due to the coupling of the dielectric tetrahedron to the incident magnetic field.

For the nonferrous materials with the relative permeability close to 1, the first resonant frequency $f_{1}$ of the dielectric spheres^1,26^ or cubes^22^ is determined by the permittivity $\varepsilon _{\mathrm{r}}$ and their effective size *L* as $f_{1} = c/L\varepsilon _{\mathrm{r}}^{1/2}$, where *c* is the speed of light in vacuum. For spheres^1,26^ with a radius *r*, there is $L = 2r$; while for cubes^22^ with a side length *l*, there is $L = 2^{1/2}l$. Here, for the dielectric tetrahedron, the effective size *L* can be estimated with $L = 0.56a$, according to the numerical simulation results shown in Supplementary Fig. S2. Therefore, the first resonant frequency of a tetrahedron can be estimated with

1 $$\begin{aligned} f_{1} = c/0.56a\varepsilon _{\mathrm{r}}^{1/2}. \end{aligned}$$

The experimental results of $f_{1} = 0.27\hbox { THz}$ for the transmission spectra of both the upright and the upside-down tetrahedrons match well with Eq. (1), and the value of *L* is slightly smaller than the diameter of its volume-equivalent sphere, which is 0.6*a*.

In addition, the transmission spectra of the tetrahedrons are independent of their orientation, which is different from the feature of the cubes^27^. When a upright tetrahedron is rotated around the *x* axis with the incident angle ${\upvarphi }$ changing from $-54.7^{\circ }$ to $70.5^{\circ }$, the simulation results of the transmission spectra are approximately the same, as shown in Supplementary Fig. S3, where the upright and the upside-down tetrahedrons correspond to two typical cases with ${\upvarphi } = 0^{\circ }$ and ${\upvarphi } = 70.5^{\circ }$, respectively. The asymmetric geometry with respect to the plane perpendicular to the incident wave has no effect on the transmission spectra of the tetrahedrons.

However, the orientation of the tetrahedron affects the reflection spectra. For the upright tetrahedron, the magnetic dipole resonance enhances at its first magnetic resonant frequency $f_{1}$, as shown in Fig. 2c. But for the upside-down tetrahedrons, no resonance is excited at $f_{1}$, because both the magnetic and the electric fields are weak inside the tetrahedron, as shown in Fig. 2d. In addition, the resonances at $f_{2}$ for all four cases are proved to be electric resonances, according to the simulation results as shown in Supplementary Fig. S4. It indicates that the disappear of the resonance at $f_{1}$ is attributed to the cancellation of the magnetic dipole resonance. The reason is explained by the superposition of two magnetic dipole resonances, which are induced by the incident wave passing it and the reflected wave passing its flipped counterpart, respectively, as shown in Fig. 2e,f. As the frequencies to excite the magnetic dipole resonances at the centroid in an upright tetrahedron and its upside-down counterpart are both $f_{1}$, the phase difference between the two magnetic dipoles can lead to the interference between them.

Here we notice that the centroid-substrate distances of the upright and the upside-down tetrahedrons, $D_{0}$ and $D_{1}$, are different, as shown in Fig. 2d. As the reflected magnetic field has a near-zero phase change at the surface of the copper substrate, i.e. a perfect electric conductor, the phase difference between the two magnetic dipoles is determined by the wave path difference *s* at the centroid between the reflected wave and the incident wave. For the upright tetrahedron, there is $s = 2D_{0} = 2(d + h_{\mathrm{c}}) = 224\,{\upmu }\hbox {m} \approx \lambda _{1}/5$, which indicates that the two magnetic dipole resonances are approximately in phase as a constructive interference. In contrast, for the upside-down tetrahedron, there is $s = 2D_{1} = 2(d + h_{\mathrm{c}} + \Delta D) = 514\,{\upmu }\hbox {m} \approx \lambda _{1}/2$, which satisfies the condition for the destructive interference with a phase difference of $\pi$, as shown in Fig. 2f. The destructive interference results in the cancellation of the two magnetic dipoles, which explains the disappearance of the first magnetic resonance in the upside-down tetrahedron.

### Controlled interference between two magnetic dipoles in an upright tetrahedron

**Figure 3:**
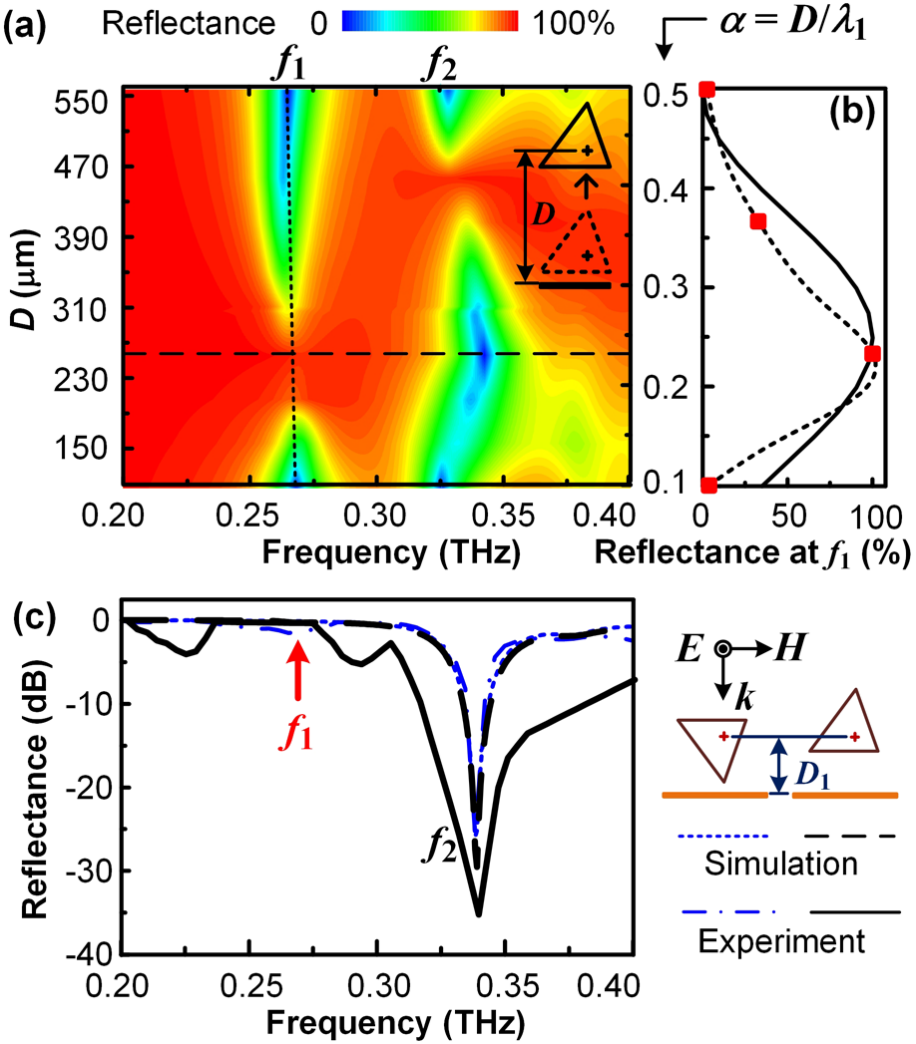
Effect of the centroid-substrate distance *D* on the terahertz reflection spectra of an upright tetrahedron. (**a**) Simulation results of the reflection spectra with the variation of *D*. The reflectance curves of the dotted and the dashed lines in (**a**) correspond to the dotted and the dashed lines in (**b**,**c**), respectively. (**b**) The magnitude variation of the reflectance at $f_{1}$ with the normalized centroid-substrate distance $\alpha$. The continuous and the dotted lines represent the simulation result and the theoretical prediction, respectively, while the square symbols represent the experimental results. (**c**) The reflection spectra of the upright tetrahedron with the same centroid-substrate distance $D = D_{1} = 257\,{\upmu }\hbox {m}$ as the upside-down tetrahedron in Fig. 1g. The black dashed line and the continuous line represent the simulation and the experimental reflection spectra of the upright tetrahedron, respectively. The blue dotted line and the dash-dotted line are the simulation and the experimental reflection spectra of the upside-down tetrahedron for comparison, respectively.

The effect of the interference on the reflection spectra is further verified with an increasing *D* by raising the upright tetrahedrons, as shown in the inset in Fig. 3a. A normalized centroid-substrate distance $\alpha$ is defined as $\alpha = D/\lambda _{1}$, so $\alpha = 0.25$ represents a phase difference of $\pi$ for destructive interference while $\alpha = 0.5$ represents a phase difference of $2\pi$ for a constructive interference. According to the simulation results, although the electric resonant frequency $f_{2}$ is sensitive to the centroid-substrate distance *D* as reported in the literature^14,19^, the magnetic resonant frequency $f_{1}$ is almost unchanged with the increase of *D* from $110\,{\upmu }\hbox {m}$ ($\alpha = 0.1$) to 550 ${\upmu }\hbox {m}$ ($\alpha = 0.5$), as shown with the dotted line in Fig. 3a. And the reflectance at $f_{1} = 0.27\hbox { THz}$ increases from $\thicksim$ 0 to $\thicksim$ 100 % and then decreases back to $\thicksim$ 0 % with the increase of $\alpha$ from 0.1 to $\thicksim$ 0.25 then to 0.5 as expected, as shown with the dotted line in Fig. 3b. The continuous line represents the theoretic prediction of the reflectance change at $f_{1}$ due to the phase differences as $R(f_{1}) = [1 - \hbox {cos}(4\pi \alpha )]/2$, while the square symbols represent the experimental results. It confirms that the interference between the two magnetic dipoles induced by the indent wave and the reflected wave determines the magnitude of the terahertz reflection of the copper-substrate-coupled tetrahedrons at the first magnetic resonance.

In the experiments, a pure PDMS film with a specific thickness is inserted under the tetrahedron-embedded PDMS film to raise *D* to the desired value. The results of the reflection spectra for $\alpha = 0.1$ have been shown in Fig. 1f2, while the results for $\alpha = 0.37$ and 0.5 are shown in Supplementary Fig. S5. The reflection spectra for $\alpha = 0.23$ are similar to the results of the upside-down tetrahedrons with the same $D_{1} = 257\,{\upmu }\hbox {m}$, as shown in Fig. 3c. Although the destructive interference in the upright tetrahedron also results in a perfect reflector at $f_{1}$, the total thickness of the surface structure with the upright tetrahedrons is $\thicksim$ 30 % larger than that with the upside-down tetrahedrons.

## Discussion

In summary, we fabricate micro $\hbox {ZrO}_{2}$ ceramic tetrahedrons and use them to investigate the role of the asymmetric geometry of micro dielectric particles in response to the terahertz EM waves. We find that the orientation of the tetrahedrons will not affect the transmission spectra. However, the first magnetic resonance disappears when the tetrahedron is flipped upside down from its rest position on a metallic substrate. The reason is attributed to the destructive interference between the two magnetic dipoles induced respectively by the incident and the reflected wave, which diminishes the magnetic resonance in the upside-down tetrahedron. According to the fact that the terahertz response of the dielectric particles can be altered by the interference between the magnetic dipole resonances, it is interesting to find that the same asymmetric particle can be used as both a perfect absorber and a perfect reflector. It brings new insights into the design of the materials with 3D building blocks to realize more interesting and exotic terahertz properties.

## Methods

### Fabrication procedure of $\hbox {ZrO}_{2}$ ceramic tetrahedrons

**Figure 4:**
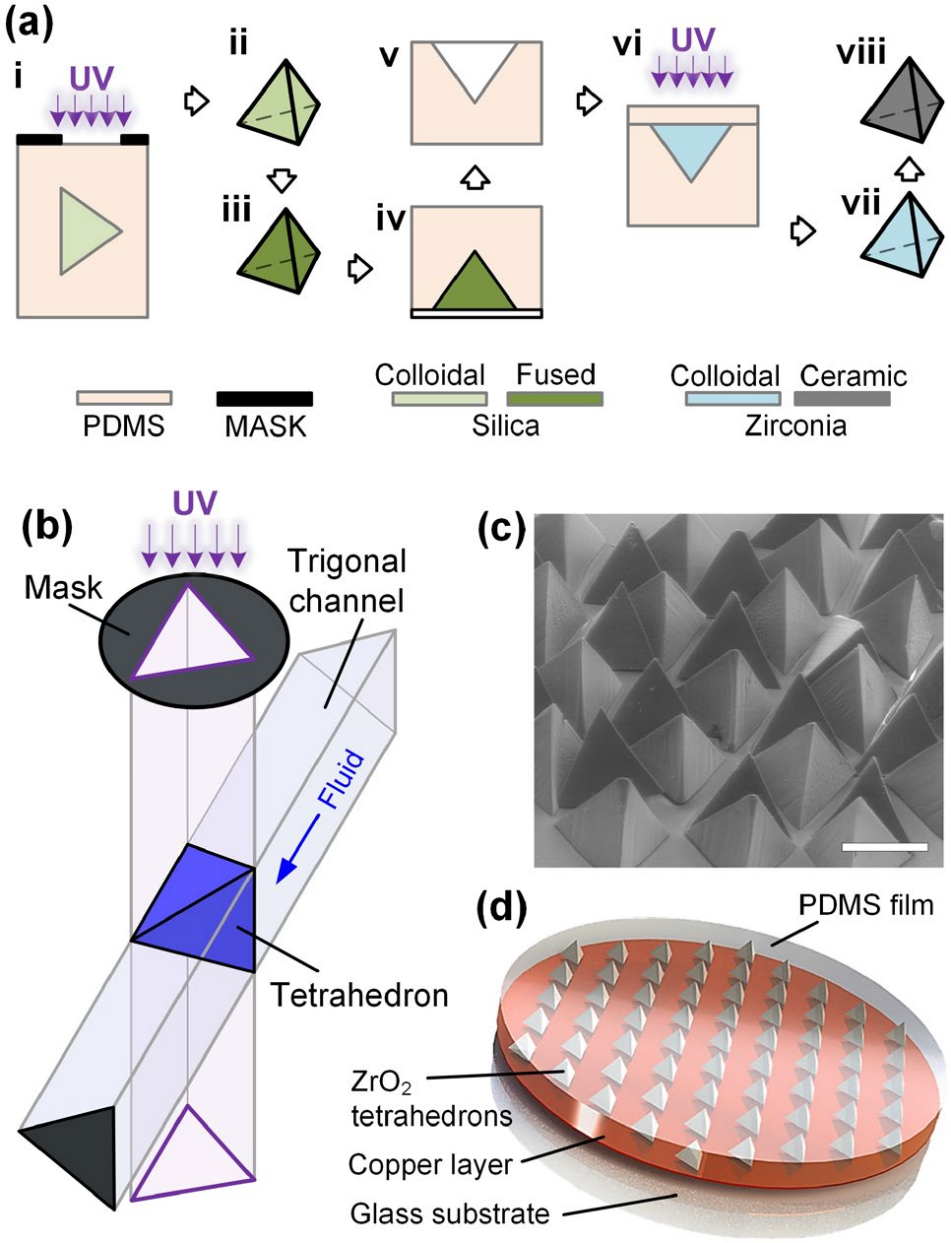
The fabrication of the $\hbox {ZrO}_{2}$ ceramic tetrahedrons. (**a**) Fabrication procedure. i: The orthogonal projection method to fabricate silica-colloidal tetrahedrons. ii: The silica-colloidal tetrahedrons after UV polymerization. iii: The fused silica tetrahedrons after sintering. iv: Making a negative PDMS mold using the fused silica tetrahedrons as templates. v: The negative PDMS mold. vi: UV polymerization of the $\hbox {ZrO}_{2}$ colloidal tetrahedrons in the mold. Step vii: The polymerized $\hbox {ZrO}_{2}$ colloidal tetrahedrons. viii: $\hbox {ZrO}_{2}$ ceramic tetrahedrons after sintering. (**b**) The schematics of the orthogonal projection method to fabricate UV polymerized tetrahedrons. (**c**) The SEM image of the fabricated silica colloidal tetrahedrons. The scale bar is 1 mm. (**d**) The schematics of the sample for the terahertz response measurement. The $\hbox {ZrO}_{2}$ tetrahedrons are embedded in a PDMS film. The PDMS film is reusable in the terahertz measurements, and can be reversibly bonded on a copper film in the reflection measurements.

The $\hbox {ZrO}_{2}$ tetrahedrons used in the experiments are fabricated with a replica casting technique^18^ from a mold of negative replica of silica tetrahedron templates, as shown in Fig. 4a. First, the silica tetrahedron templates are fabricated with a orthogonal projection method (i), the detail schematics of which is shown in Fig. 4b. Silica nanoparticles are well dispersed in an ultraviolet(UV)-curable fluid^28^, and the resultant colloidal suspension is injected into a PDMS microchannel with a triangular cross section. Then a photo mask with a triangular window is aligned with the PDMS channel, and a polymerized colloidal tetrahedron is made upon a UV exposure for 400 ms. The resultant tetrahedron is pushed away from the exposure site by the flowing fluid in the channel, so that another UV-exposure can be proceed to generate a new colloidal tetrahedron. The generation process is consecutive, and the resultant tetrahedrons are monodisperse, as shown in Fig. 4c. The orthogonal projection method for fabricating micro tetrahedrons has much higher production rate and much higher resolution than the 3D printing method^18^. After the polymerization, colloidal tetrahedrons are washed and dried (ii). Then, they are sintered to form the fused-silica tetrahedrons (iii).

Second, the silica tetrahedron templates serve as a positive shape to make a negative PDMS mold (iv and v). A ceramic slurry with $\hbox {ZrO}_{2}$ powder in a UV-curable suspension is then filled into the negative PDMS mold, and a PDMS slab is covered on top (vi). Then, each side of the bulk mold is exposure to UV for 15 s to polymerize the tetrahedrons. The shape of the polymerized $\hbox {ZrO}_{2}$ colloidal particle (vii) is controlled by the cavities on the negative PDMS mold. After the polymerized $\hbox {ZrO}_{2}$ tetrahedrons are dried, they are sintered to form the $\hbox {ZrO}_{2}$ ceramic tetrahedrons (viii) with tetragonal crystal structure.

### Precursor fluids for silica tetrahedrons

A precursor suspension is prepared by mixing 22 wt.% poly(ethylene glycol) diacrylate (PEG-DA, Mn = 700 g/mol, Sigma-Aldrich), 74 wt.% LUDOX CL-X colloidal silica (45 wt.% suspension in H_2_O, Sigma-Aldrich) and 4 wt.% 2-Hydroxy-2-methyl-propiophenone (PI, Darocur 1173, Sigma-Aldrich). After synthesis, the tetrahedrons are collected and washed with 0.5 v/v % Tween-20 (Sigma-Aldrich) solution in water.

### Temperature conditions for sintering $\hbox {SiO}_{2}$ tetrahedrons

The $\hbox {SiO}_{2}$ colloidal tetrahedrons are exposed in ambient environment and dried at room temperature for 24 h, and then, are sintered in the oven. The temperature in the oven is first increased to $600\,^{\circ }\hbox {C}$ at the rate of $0.5\,^{\circ }\hbox {C}/\hbox {min}$, and maintained at $600\,^{\circ }\hbox {C}$ for 2 h for the polymer binder to be to removed completely. Then, the temperature is increased to $1150\,^{\circ }\hbox {C}$ at the rate of $1\,^{\circ }\hbox {C}/\hbox {min}$, and maintained at $1150\,^{\circ }\hbox {C}$ for 2 h to consolidate the $\hbox {SiO}_{2}$ nanoparticles before furnace cooling. Fused silica tetrahedrons are obtained after sintering.

### Precursor fluids for $\hbox {ZrO}_{2}$ tetrahedrons

$\hbox {ZrO}_{2}$ dispersion is made by dispersing the commercially available yttria-stabilized zirconia (YSZ) ceramic powder (Shanghai Yaoyi alloy material Co., Ltd., China, 1 ${\upmu }\hbox {m}$; purity $\ge$ 99.99 wt.%) into the deionized water of the same volume. 0.5 wt.% SN-dispersant 5040 (Yixing Kexin Chemical Co., Ltd., China) is added based on the powder as dispersant. The composite suspension is ball-milled for 24 h in a planetary ball mill (rotating at 450 r/min) containing $\hbox {ZrO}_{2}$ balls. Acrylamide (AM, Shanghai Macklin Biochemical Co., Ltd., AR), N,N$^{\prime }$-methylenebisacrylamide (MBAM, Shangdong Xiya chemical Co., Ltd., AR) and PI are used as monomer, cross-linker and photoinitiator, respectively. AM is 9 w/v % based on $\hbox {ZrO}_{2}$ dispersion, MBAM is 1/6 of AM, the PI is 4 v/v % of the whole solution.

### Temperature conditions for sintering $\hbox {ZrO}_{2}$ tetrahedrons

The $\hbox {ZrO}_{2}$ colloidal tetrahedrons are dried for 24 h at room temperature, and then, sintered in the oven. The temperature in the oven is first increased stepwisely to $600\,^{\circ }\hbox {C}$ at the rate of $1\,^{\circ }\hbox {C}/\hbox {min}$, and maintained at $114\,^{\circ }\hbox {C}$, $235\,^{\circ }\hbox {C}$, $374\,^{\circ }\hbox {C}$, $495\,^{\circ }\hbox {C}$, $600\,^{\circ }\hbox {C}$ for 1 h each, respectively, to remove the polymer binder gradually and avoid the generation of cracks. Then, the temperature is increased to $1550\,^{\circ }\hbox {C}$ at the rate of $5\,^{\circ }\hbox {C}$ /min, and maintained at $1550\,^{\circ }\hbox {C}$ for 2 h to consolidate $\hbox {ZrO}_{2}$ nanoparticles before furnace cooling. Zirconia ceramic tetrahedrons with tetragonal crystal structure are obtained after sintering.

### Preparing tetrahedron-embedded PDMS film for the measurement of terahertz response

A pattern of hexagonal lattice is made on the photoresist layer on a glass substrate using photolithography method. The $\hbox {ZrO}_{2}$ ceramic particles are placed in the hexagonal lattice. After the array of tetrahedrons is prepared, the mixture of the PDMS base fluid and the crosslinking oligomers with a ratio of 10:1 is poured on the array^29^. With the aid of the spacers, which has a fixed thickness, the thickness of the tetrahedron-embedded PDMS film is controlled, and subsequently the separation between the tetrahedrons and the PDMS surface is controlled. After the polymerization at $70\,^{\circ }\hbox {C}$ for 90 min, the tetrahedron-embedded PDMS film is obtained, which is reusable in all the experiments, as shown in Fig. 4d. As both sides of the tetrahedron-embedded PDMS film can be reversely bonded on the copper layer, the upside-down flip of the tetrahedrons can be realized by flipping the PDMS film.

### Simulation of the terahertz response

Numerical simulations of the terahertz responses of the dielectric tetrahedrons are performed with the commercial finite difference time-domain and frequency-domain package CST MICROWAVE STUDIO. The boundary conditions for both of the transmission mode and the reflection mode are set as unit cell for *x* direction, unit cell for *y* direction, and open for *z* direction. The unit cell models of the transmission mode and the reflection mode are shown in Supplementary Fig. S1(a) and (b), respectively. Each unit cell has one dielectric tetrahedron inside as the resonator, while in the reflection mode, there is a copper film under the tetrahedron with a separation of $40\,{\upmu }\hbox {m}$ to its bottom surface. Two floquet ports are chosen for the default Zmax excitation source; tetrahedral mesh is chosen for the frequency domain solver; while the range of a broadband frequency sweep is 0–0.7 THz in the simulation. The S-parameter error threshold is set to 0.01.

### Experimental measurements of the terahertz response

The terahertz transmission and reflection spectra are experimentally measured with the terahertz time-domain spectrometer (TAS7500TS, ADVANTEST, Japan). The dry air unit is turned on to eliminate moisture interference. The beam paths of the transmission module and the reflection module of the spectrometer are shown schematically in Supplementary Fig. S6(a) and (b), respectively. The beam spot size is approximately 5 mm. In the reflection module, there are angles of incidence and reflection to prevent interference between the assembly components. The angles are both $11^{\circ }$, while they are approximately considered as normal incidence and reflection. The transmission spectrum of the dry air environment is measured to normalize the features in the transmission, while the transmission spectrum of a pure PDMS film with a thickness of $370\,{\upmu }\hbox {m}$ is measured as the background for the transmission measurements of the tetrahedron-embedded PDMS film. The reflection spectrum of a standard metal block is measured to normalize the features in the reflection experiments, while the reflection spectrum of the pure PDMS film on the copper substrate is measured as the background for the reflection measurements of the tetrahedron-embedded PDMS film on the copper substrate. The measured backgrounds are shown in Supplementary Fig. S7. For the reflection measurements, we set the time-window for FFT calculation, where the start time and the stop time are set to 0 psec and 131 psec, respectively, for both of the sample and the background.

## Supplementary information

Supplementary Information.

## References

[1] Soukoulis CM, Wegener M (2011). Past achievements and future challenges in the development of three-dimensional photonic metamaterials. Nat. Photonics.

[2] Kristensen A (2017). Plasmonic colour generation. Nat. Rev. Mater..

[3] Jahani S, Jacob Z (2016). All-dielectric metamaterials. Nat. Nanotechnol..

[4] Cai W, Chettiar UK, Kildishev AV, Shalaev VM (2007). Optical cloaking with metamaterials. Nat. Photonics.

[5] Schurig D (2006). Metamaterial electromagnetic cloak at microwave frequencies. Science.

[6] Kim Y (2018). Robust control of a multifrequency metamaterial cloak featuring intrinsic harmonic selection. Phys. Rev. Appl..

[7] Ma Z (2016). Terahertz all-dielectric magnetic mirror metasurfaces. ACS Photonics.

[8] Yu P (2019). Broadband metamaterial absorbers. Adv. Opt. Mater..

[9] Radi Y, Simovski CR, Tretyakov SA (2015). Thin perfect absorbers for electromagnetic waves: theory, design, and realizations. Phys. Rev. Appl..

[10] Moitra P (2015). Large-scale all-dielectric metamaterial perfect reflectors. ACS Photonics.

[11] Corbitt SJ, Francoeur M, Raeymaekers B (2015). Implementation of optical dielectric metamaterials: a review. J. Quant. Spectrosc. Radiat. Transf..

[12] Wang X, Diaz-Rubio A, Li H, Tretyakov SA, Alu A (2020). Theory and design of multifunctional space-time metasurfaces. Phys. Rev. Appl..

[13] Prinz VY (2017). Terahertz metamaterials and systems based on rolled-up 3d elements: designs, technological approaches, and properties. Sci. Rep..

[14] Zheng P, Kasani S, Wu N (2019). Converting plasmonic light scattering to confined light absorption and creating plexcitons by coupling a gold nano-pyramid array onto a silica-gold film. Nanoscale Horizons.

[15] Ferguson B, Zhang XC (2002). Materials for terahertz science and technology. Nat. Mater..

[16] Manjappa M, Singh R (2020). Materials for terahertz optical science and technology. Adv. Opt. Mater..

[17] Torquato S, Jiao Y (2009). Dense packings of the platonic and archimedean solids. Nature.

[18] Mirkhalaf M, Zhou T, Barthelat F (2018). Simultaneous improvements of strength and toughness in topologically interlocked ceramics. Proc. Natl. Acad. Sci. U.S.A..

[19] Moreau A (2012). Controlled-reflectance surfaces with film-coupled colloidal nanoantennas. Nature.

[20] Gao J, Lan C, Zhao Q, Li B, Zhou J (2018). Experimental realization of mie-resonance terahertz absorber by self-assembly method. Opt. Express.

[21] Watanabe M, Kuroda S, Yamawaki H, Shiwa M (2011). Terahertz dielectric properties of plasma-sprayed thermal-barrier coatings. Surf. Coat. Technol..

[22] Liu X, Zhao Q, Lan C, Zhou J (2013). Isotropic mie resonance-based metamaterial perfect absorber. Appl. Phys. Lett..

[23] Roy Chowdhury D (2011). Dynamically reconfigurable terahertz metamaterial through photo-doped semiconductor. Appl. Phys. Lett..

[24] Liu X, Fan K, Shadrivov IV, Padilla WJ (2017). Experimental realization of a terahertz all-dielectric metasurface absorber. Opt. Express.

[25] He X, Liu F, Lin F, Shi W (2019). Investigation of terahertz all-dielectric metamaterials. Opt. Express.

[26] Zhao Q, Zhou J, Zhang F, Lippens D (2009). Mie resonance-based dielectric metamaterials. Mater. Today.

[27] Zeng X, Zhang G, Xi X, Li B, Zhou J (2019). Terahertz transmission of square-particle and rod structured TbFeO$$_{3}$$ metamaterials. Mater. Lett..

[28] Shepherd RF (2008). Stop-flow lithography of colloidal, glass, and silicon microcomponents. Adv. Mater..

[29] Duffy DC, McDonald JC, Schueller OJ, Whitesides GM (1998). Rapid prototyping of microfluidic systems in poly(dimethylsiloxane). Anal. Chem..

