# Supplementary material for: Dielectric tetrahedrons as terahertz resonators switched from perfect absorber to reflector
Source: Sci Rep. 2020 Oct 13;10:17134. doi: 10.1038/s41598-020-74252-0 (PMC7553947; doi:10.1038/s41598-020-74252-0)
Supplement: Supplementary file 1 — Supplementary Information. [file 41598_2020_74252_MOESM1_ESM.pdf]

Dielectric tetrahedrons as terahertz resonators switched from perfect absorber to reflector

Haosheng Chen[1,+], Chenchen Zhou[1,+], Yongjian Li[1], Shuaishuai Liang[2], and Jiang Li[2,*]

[1]State Key Laboratory of Tribology, Tsinghua University, Beijing 100084, China
[2]School of Mechanical Engineering, University of Science and Technology Beijing, Beijing 100083, China
[*]corresponding.lijiang@ustb.edu.cn
[+]these authors contributed equally to this work

ABSTRACT

Tetrahedrons are basic building blocks in natural and artificial materials, while the terahertz response of micro tetrahedrons has been little explored. Here we fabricate ceramic tetrahedrons in the subwavelength of terahertz range, and find that the three-dimensional geometry significantly affects their terahertz properties. The transmission spectra are independent of the orientation of the tetrahedrons, while the first magnetic resonance disappears in the reflection spectra when an upright tetrahedron is flipped upside down on the metallic substrate, which changes it from a perfect absorber to a perfect reflector. This is attributed to the destructive interference between two magnetic dipoles induced respectively by the incident and the reflected wave. The study brings new insights in the materials design with 3D building blocks to realize more interesting and exotic terahertz properties.

Supplementary Information

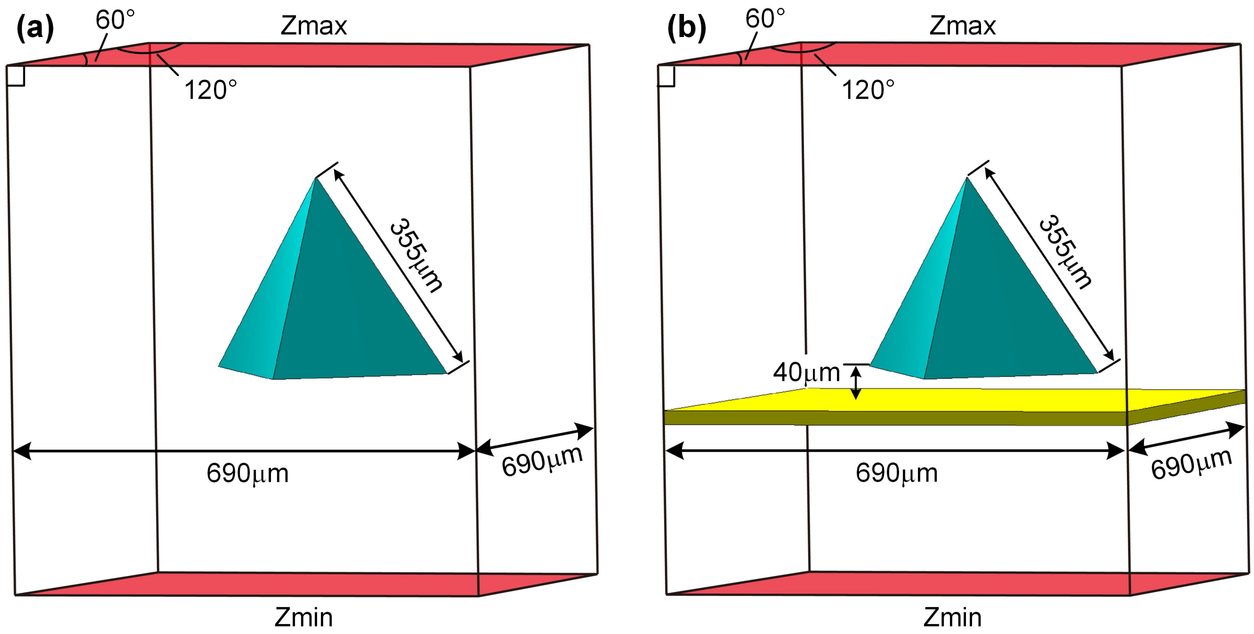

Figure S1. Unit cell models used in the numerical simulations. (a) Unit cell model for the transmission mode simulations. There is one dielectric tetrahedron inside. (b) Unit cell model for the reflection mode simulations. There is a copper film under the dielectric tetrahedron, where the separation between the bottom of the tetrahedron and the copper film is 40 μm.

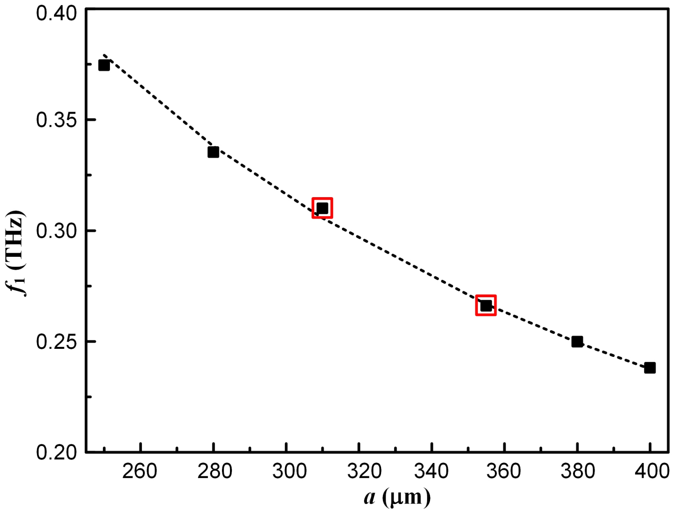

Figure S2. The dependence of the first resonant frequencies on the side length a of the ZrO_2 ceramic tetrahedrons. The filled square symbols in black are the simulation results, while the open square symbols in red are the experimental results. The dotted line is the best fit of the simulation results, which indicates $f_1 = c/0.56a\varepsilon_r^{1/2}$.

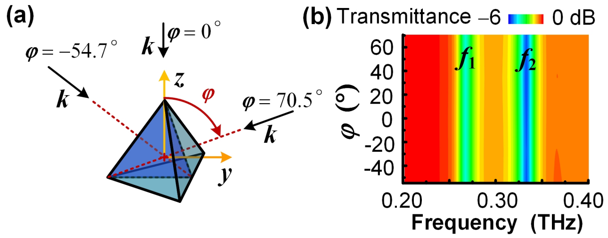

Figure S3. The independence of the transmission spectra on the incident angle. (a) The schematics of the variation of the incident angle φ. (b) The transmission spectra with different incident angles. Both the first and the second resonant frequencies, f_1 and f_2, remain the same for all the incident angles.

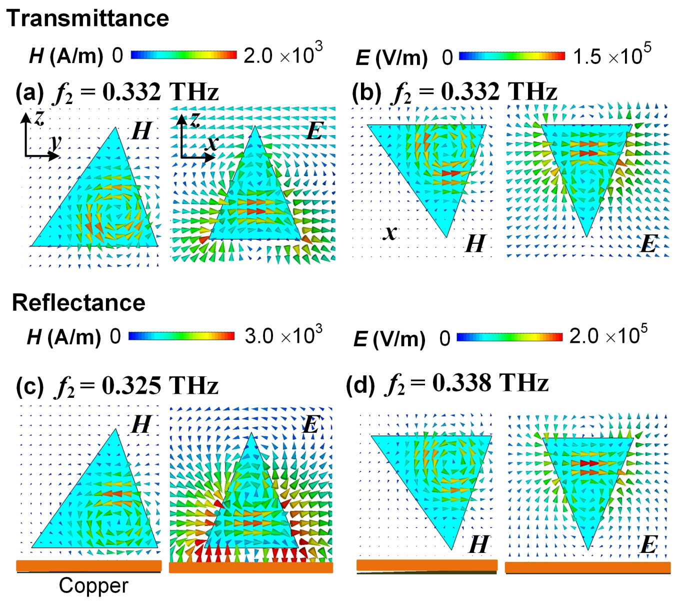

Figure S4. Electromagnetic fields at the second resonant frequency f_2, which corresponds to the electric dipole resonance. The tetrahedrons can be considered as equivalent electric dipoles at the centroid in the x-z plane, while the effective magnetic current wraps around the centroid of the tetrahedron in the y-z plane. (a) Electromagnetic fields at the second resonant frequency f_2 in Fig. 1(d2) for the transmission of an upright tetrahedron. (b) Electromagnetic fields at f_2 in Fig. 1(e2) for the transmission of an upside-down tetrahedron. (c) Electromagnetic fields at f_2 in Fig. 1(f2) for the reflection of an upright tetrahedron on a copper layer. (d) Electromagnetic fields at f_2 in Fig. 1(g2) for the reflection of an upside-down tetrahedron.

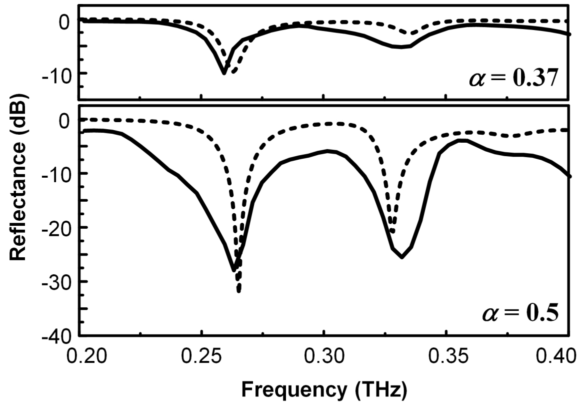

Figure S5. The reflection spectra of the upright tetrahedron on a copper substrate with the normalized centroid-substrate distance α = 0.37 and 0.5, respectively. The dotted lines represent the simulation results, while the continuous lines represent the experimental results.

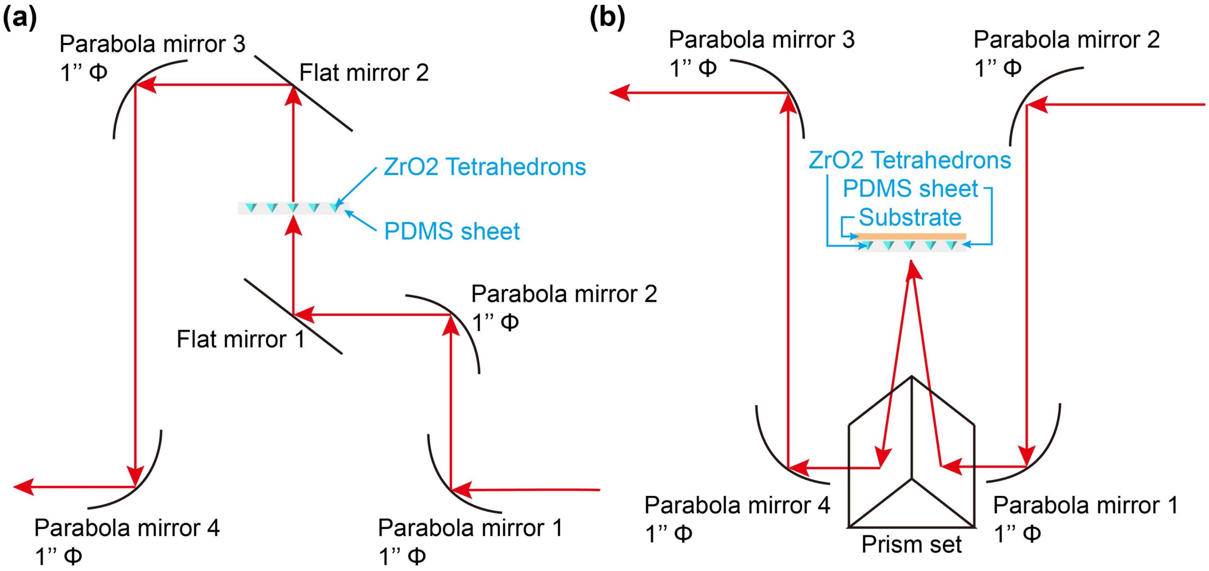

Figure S6. Beam path diagram of the terahertz wave. (a) Beam path in the transmission module. (b) Beam path in the reflection module.

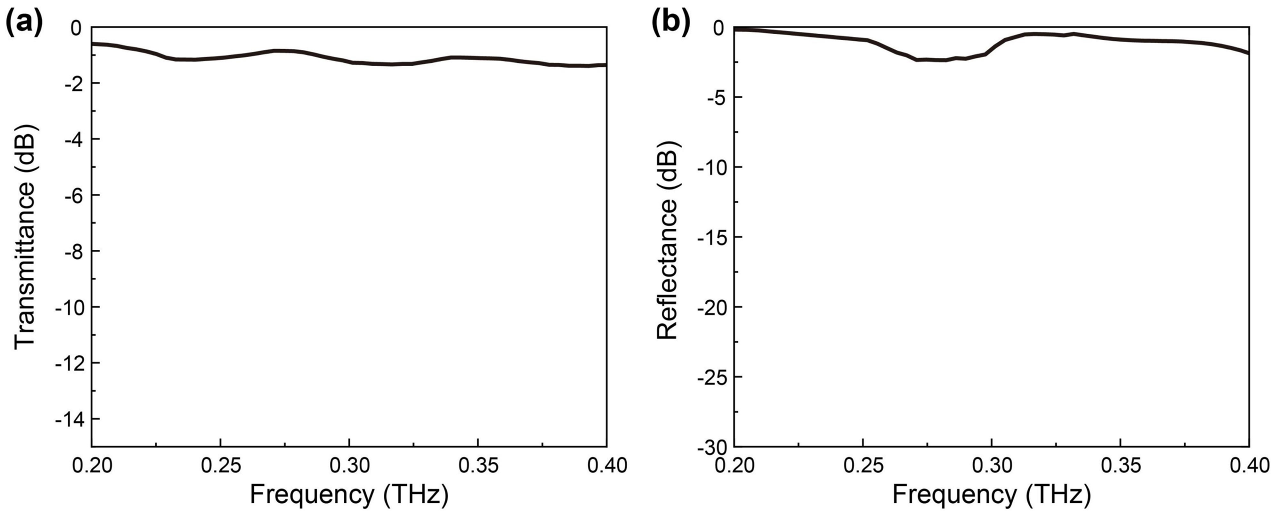

Figure S7. Background spectra in the transmission and the reflection experiments. (a) The measured spectrum of a PDMS film with a thickness of 370 μm, which is used as the background spectrum for the transmission experiments. (b) The measured spectrum of the PDMS film adhered on the copper substrate, which is used as the background spectrum for the reflection experiments. The results indicate that the PDMS film has a low loss and has no obvious peak in the range of 0.2 to 0.4 THz, so we do not consider the loss of PDMS in the experiments.
